# Prevalence and Risk Factors for Adult Pulmonary Tuberculosis in a Metropolitan City of South India

**DOI:** 10.1371/journal.pone.0124260

**Published:** 2015-04-23

**Authors:** Baskaran Dhanaraj, Mohan Kumar Papanna, Srividya Adinarayanan, Chandrasekaran Vedachalam, Vijayaraj Sundaram, Shivakumar Shanmugam, Gomathi Sekar, Pradeep Aravindan Menon, Fraser Wares, Soumya Swaminathan

**Affiliations:** 1 National Institute for Research in Tuberculosis, Chennai, India; 2 India EIS Programme, National Centre for Disease Control, New Delhi, India; 3 Global TB Programme, World Health Organization, Geneva, Switzerland; University of Delhi, INDIA

## Abstract

**Background:**

The present study measured the community prevalence and risk factors of adult pulmonary tuberculosis (PTB) in Chennai city, and also studied geographical distribution and the presence of different *M*. *tuberculosis* strains in the survey area.

**Methods:**

A community-based cross sectional survey was carried out from July 2010 to October 2012 in Chennai city. Prevalence of bacteriologically positive PTB was estimated by direct standardization method. Univariate and multivariate analyses were carried out to identify significant risk factors. Drug susceptibility testing and spoligotyping was performed on isolated *M*. *tuberculosis* strains. Mapping of PTB cases was done using geographic positioning systems.

**Results:**

Of 59,957 eligible people, 55,617 were screened by X-ray and /or TB symptoms and the prevalence of smear, culture, and bacteriologically positive PTB was estimated to be 228 (95% CI 189–265), 259 (95% CI 217–299) and 349 (95% CI 330–428) per 100,000 population, respectively. Prevalence of smear, culture, and bacteriologically positive PTB was highest amongst men aged 55–64 years. Multivariate analysis showed that occurrence of both culture and bacteriologically positive PTB disease was significantly associated with: age >35 years, past history of TB treatment, BMI <18.5 Kgs/m^2^, solid cooking fuel, and being a male currently consuming alcohol. The most frequent spoligotype family was East African Indian. Spatial distribution showed that a high proportion of patients were clustered in the densely populated north eastern part of the city.

**Conclusion:**

Our findings demonstrate that TB is a major public health problem in this urban area of south India, and support the use of intensified case finding in high risk groups. Undernutrition, slum dwelling, indoor air pollution and alcohol intake are modifiable risk factors for TB disease.

## Introduction

India has more tuberculosis (TB) cases annually than any other country globally, with an estimated disease prevalence of 256/100,000 population, incidence of 185/100,000 and deaths of 26/100,000. Nationwide annual risk of TB infection (ARTI) surveys had estimated an ARTI of 1.5% (in 2000–2001) and 1.1% (in 2009–2010), with an average annual decline of 3.6%. The Revised National Tuberculosis Control Programme (RNTCP) provides free diagnosis and treatment to all TB patients in the public sector, and has successfully treated over 15 million patients in the past 10 years. Currently under RNTCP, any person presenting with a cough of more than two weeks is screened for pulmonary TB (PTB) by two sputum smear examinations, (one spot and one overnight sample) at designated microscopic centres. Treatment of TB patients is based on the internationally recommended directly observed treatment short course (DOTS) strategy. Newly diagnosed smear positive TB patients are treated with a 6-month thrice weekly regimen (Category I); 2 months isoniazid (H) rifampicin (R) pyrazinamide (Z) ethambutol (E) (HRZE)/4 months HR, and retreatment patients with a 8-months thrice weekly regimen (Category II); 2 months HRZES (S streptomycin)/1 month HRZE/5 months HRE [1].

In high TB endemic (>100/100,000 population) countries such as India, the World Health Organization (WHO) recommends periodic disease prevalence surveys to measure the effect of TB control measures [2]. Prevalence surveys help to evaluate the burden of disease in the community and accurately estimate the prevalence of smear and culture positive PTB, which is not feasible under routine programmatic conditions. Additionally, such surveys provide unique opportunities to explore the interactions between TB disease and its sociocultural and environmental determinants. Four previous prevalence surveys of PTB conducted by the National Institute for Research in Tuberculosis (NIRT) over a period of 10 year post−DOTS implementation under RNTCP in a peri−urban area close to the city of Chennai (in Tiruvallur district), south India showed an initial decreasing trend, followed by an increase in prevalence [3]. The survey reported here aimed to estimate the prevalence of pulmonary TB and study its determinants in the Chennai metropolitan area, to explore the distribution of *Mycobacterium tuberculosis* strain types, and to document the spatial distribution of TB cases detected by the survey.

## Materials and Methods

The survey was carried out from July 2010 to October 2012 amongst individuals aged ≥15 years in randomly selected wards (clusters) of Chennai city. A ‘ward’ is the smallest administrative unit in the city, with a population of approximately 45,000. A sample size of 53,142 was estimated by assuming a disease prevalence of 400/100,000 population with a precision of 20%, a design effect of 2 and 10% dropout. A cluster sampling design with replacement was adopted, where wards were the clusters, and it was decided to survey 600 individuals from each selected ward. To achieve the required sample size, wards were selected based on population proportional to size. A total of 100 wards were selected from the 155 wards of the Chennai City Corporation. A ward consists of both slum (defined as a compact area of at least 300 population or about 60–70 households of poorly built congested tenements, in unhygienic environment usually with inadequate infrastructure and lacking in proper sanitary and drinking water facilities) [4] and non-slum areas. Based on the 2001 census data, the slum population in Chennai city during 2001 was estimated to be 1,079,414 of a total population of 4,629,462. The annual growth rates from 1991 to 2001 were 2.81% for slum and 1.09% for non-slum populations respectively [5]. Based on the annual growth rates, the slum population was estimated to be 30% of the total population in 2010. In order to ensure proper representation from both areas, 200 and 400 individuals were selected from slum and non-slum areas respectively in each ward.

To achieve the required sample size from each ward, streets were randomly selected and individual households were enrolled. In case the required number of individuals were not obtained in a selected street, an adjacent street was selected until the required number was achieved. In case the same ward was selected for the second time, different streets were selected. All the households in the selected streets were registered by door-to-door census and subjects aged ≥15 years were questioned about chest symptoms suggestive of TB disease. Mass Miniature Radiography (MMR) was done for all individuals and the radiograph was read independently by two readers. In cases of disagreement, the radiograph was read by a third reader. For those with an abnormal chest radiograph or chest symptoms, two sputum samples were collected; the first on the spot and the second in the early morning of the next day. The samples were transported to the NIRT laboratory on the same day. Those individuals who were not available for examination on the day of the visit, were revisited until at least 90% of the study population had undergone investigations. The houses with smear/culture positive patients were revisited with the reports and referred to the nearest RNTCP DOTS centre. Additional data related to occupation, past history of TB treatment, alcohol consumption, tobacco smoking and fuel used were collected. The data relating to tobacco smoking and alcohol was self-reported. In case of subjects who said ‘yes’ for tobacco smoking or alcohol consumption, additional data related to smoking included age at staring the habit, type of tobacco used (Beedi, cigarette, both, others) duration in months, number of cigarettes per day (1–10,11–20,>20). Similarly, for alcohol consumption, data collected included age at starting the habit, duration in months, quantity per week (<200ml, 201–500ml, 501–1000ml and >1000ml). Height and weight of individuals was also measured. MMR examinations were however not performed in 6 wards due to logistical and administrative issues.

### Bacteriological and molecular typing methods

Direct smears were made from the collected sputum samples, followed by the setting up of *M*. *tuberculosis* culture on 2 slopes of Lowenstein Jensen medium, after NaOH decontamination and concentration by centrifugation. The slopes were incubated at 37°C and read every week up to 8 weeks [6]. Whichever specimen showed the earliest growth was subjected to drug susceptibility testing, whilst the other specimen was stored. Indirect sensitivity testing for the first line drugs isoniazid, rifampicin, ethambutol and streptomycin was carried out. Resistance to H, E and R was determined by the minimal inhibitory concentration method (MIC) and to S by the Resistance Ratio (RR) method. MICs of ≥ 5, 8 or 128μg/ml were defined as resistance to H, E and R respectively, and a RR ≥ 8 μg/ml was considered as resistance to S [7].

Spoligotyping was performed using a standard protocol [8] and the patterns were matched with SpolDB4 and SITVIT database. The spoligotype patterns were matched with the location of patients to look for any clustering.

### Geo coding of TB cases

All bacteriologically positive TB patients detected by the survey were mapped (after all the results were made available) using Global Positioning Systems (GPS) devices to see their spatial distribution. The map was developed by use of ArcGIS version 9.0 (Licenced to our sister institute Vector Control Research Centre (ICMR) Puducherry, India) using the geo referenced data.

### Statistical methods

Double data entry was done using Datastar package and analysed using SPSS 14.0 software (SPSS Inc., Chicago, IL, USA). Regular data quality checks were carried out on 20% of data by the statistical team. For estimation of prevalence in the community the number of positives were adjusted for non-coverage by MMR and sputum collection, which were stratified based on age and gender i.e. If *Xi* is the number of eligible persons in the *i*th subgroup (by sex and by age), *xi* is the number assessed (by radiography), *Si* is the number eligible for sputum collection (due to abnormal radiograph and/or presence of chest symptoms), *si* is the number with a sputum examination and *fi* is the number with a positive finding (culture/smear), the total number of positives in the *i*th subgroup is conventionally estimated as (*fi/si*) (*Si/xi*) *Xi* = *Ci*, i.e., assuming that the findings in those not examined would be the same as in those examined [9]. Subsequently, overall estimates of smear, culture-positive and bacteriologically positive tuberculosis for 1) males, 2) females, and 3) both sexes combined at each age and all ages, were obtained by the appropriate pooling of categories.

Multiple imputation for missing data: To correct for bias introduced by incompleteness of data (particularly those 6 wards in which X ray was not done), multiple missing value imputation [10, 11] was used for all individuals: a) without a field chest X-ray result and/or symptom screening, which includes all individuals who did not participate in the survey; b) eligible for sputum examination but whose status as a pulmonary TB case was unknown due to missing smear and/or culture results; c) ineligible for sputum examination, but with a X-ray reading that was suggestive of TB, whose status as a pulmonary TB case was thus unknown. Multiple imputation for missing values was implemented under the assumption that data were missing at random. Twenty imputed data sets were created and a logistic model was used to estimate the TB prevalence based on the pooled dataset [11]. This method allows for both clustering in the sampling design and the uncertainty introduced by imputation of missing values when estimating the 95% CI (Confidence Intervals) for the prevalence of pulmonary TB.

Univariate and multivariate analysis was performed to look for any association between TB and risk factors such as locality (slum or non-slum), age, gender, BMI, smoking, number of cigarettes/ beedis smoked per day, alcohol use, quantity of alcohol consumed per week, type of cooking fuel used, and history of previous TB treatment.

### Ethical considerations

The study was approved by the Institutional Ethics Committee at National Institute for Research in Tuberculosis, Chennai vide IEC No: 2009001. The field staff approached eligible individuals in the community and explained the procedures and risks and benefits of the study in the local language. Written informed consent was obtained from all individuals willing to participate. For participants between 15–18 years, assent was obtained from the individuals and written informed consent was obtained from parents/ guardians/caretakers/next to kin. Children below 15 years were not included in the study.

## Results

A total of 100 wards were covered in this survey. In these, a total of 59,957 eligible individuals ≥15 years of age were registered of whom 30,250 (50.5%) were males. Of these, 55,617 (93%) were screened by MMR and/or symptom screening and 50,976 (85%) by MMR alone. Of the 6,139 people eligible for sputum, 4,565 had chest symptoms suggestive of TB, 1,361 had an abnormal MMR, and 213 had a previous history of TB treatment (with no symptoms or abnormal MMR). Of these, 5,373 (88%) gave sputum samples out of which 111, 126 and 170 individuals were found to be smear positive (An individual with at least one sputum specimen positive for acid fast bacillus (AFB) on smear microscopy, irrespective of culture result), culture positive (An individual with at least one sputum specimen positive for *M*. *tuberculosis* on culture, irrespective of smear result) and bacteriologically positive (An individual with at least one sputum specimen positive for AFB on microscopy and/or *M*. *tuberculosis* on culture) [12] respectively.

**Fig 1:**
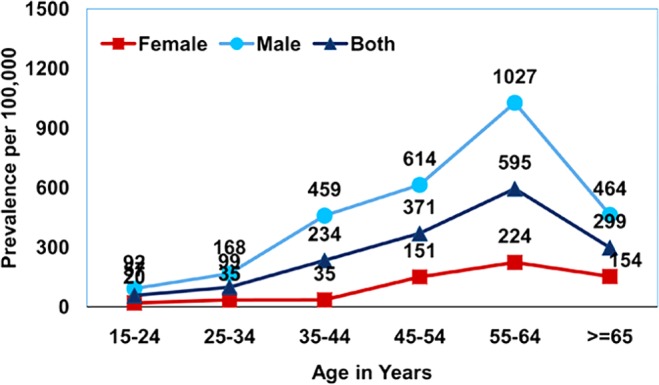
Age and sex wise prevalence of smear positive pulmonary TB.

**Fig 2:**
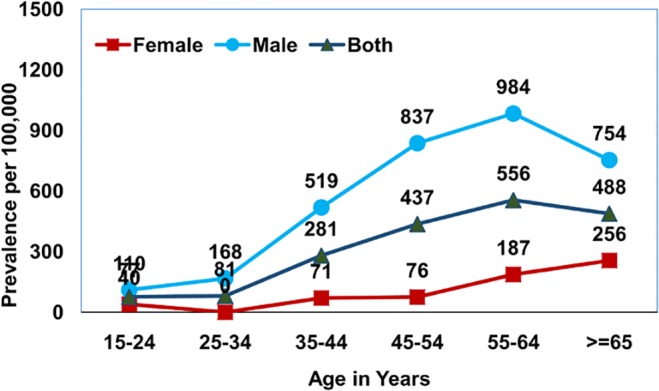
Age and sex wise prevalence of culture positive pulmonary TB.

**Fig 3:**
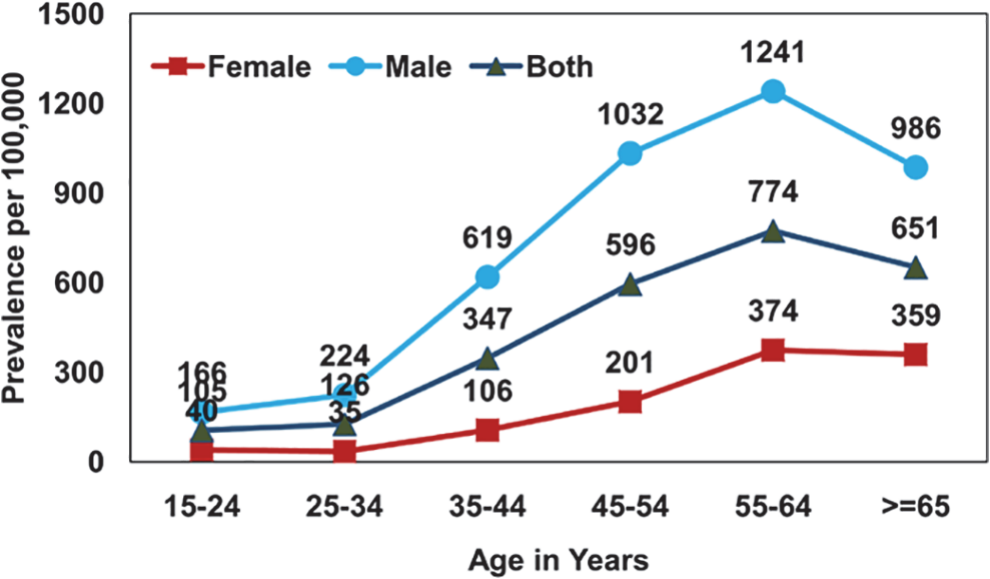
Age and sex wise prevalence of bacteriologically positive pulmonary TB.

The prevalence of smear positive PTB was estimated to be 228 (95% CI 189–265) per 100,000 population, culture positive PTB to be 259 (95% CI 217–299) and bacteriologically positive PTB to be 349 (95% CI 330–428). Those in the age group of 55 years and above had a very high prevalence of smear positive PTB estimated at 470 (95% CI 338–601), for culture positive PTB 527 (95% CI 388–666) and for bacteriologically positive PTB 722 (95%CI 605–942), Table 1. A consistently significantly higher prevalence was observed among males when compared to females for smear, culture and bacteriologically positive PTB across all age groups. While the maximum prevalence of bacteriologically positive PTB 1,241/100,000 was found in the age group of 55–64 years in males, in females in the same group, it was 374/100,000. It was observed that prevalence of smear, culture and bacteriologically positive TB increased with age and this trend was found to be significant (for smear positive TB: Chi square-45.3, df = 2, p value <0.0001, for culture positive TB: Chi square-57.3, df = 2, p value <0.0001: bacteriologically positive TB: Chi square-77.2, df = 2, p value <0.0001;). However, in the age group > 65 years, there was an observed decrease in prevalence which was relatively more for smear positive as compared to both culture and bacteriologically positive disease (Figs 1–3). It was observed that 8% of the smear positive cases turned out to be non-tuberculosis mycobacterium (NTM) on culture.

**Table 1:** Prevalence of Smear and Culture Positive Pulmonary Tuberculosis by Age and Sex (per 100,000 population).

** **	Eligible population	*Screened (%)	X ray suggestive of TB	Chest symptomatic s’	Eligible for sputum collection	No. of sputum collected (%)	No. Smr +ve#	No. Cult.+ve	No. Bact. +ve	Adjusted TB Prevalence per 100000 (95%CI)		
										Smr +ve	Cult. +ve	Bact +ve
**Age Groups **												
**15–34**	26811	24931(93)	186	1431	1792	1548(86)	17	17	25	79	79	116
										(45–113)	(45–112)	(84–170)
**35–54**	22746	20916(92)	508	1904	2517	2190(87)	53	63	82	291	346	451
										(221–361)	(270–423)	(403–585)
**≥55**	10400	9770(94)	667	1230	1830	1635(89)	41	46	63	470	527	722
										(338–601)	(388–666)	(605–942)
**TOTAL**	59957	55617(93)	1361	4565	6139	5373(88)	111	126	**170**	**228**	**259**	**349**
										**(189–265)**	**(217–299)**	**(330–428)**
**Sex**												
**Male**	30250	26943(89)	911	2642	3563	3124	91	107	135	385	452	571
										(315–455)	(377–529)	(487–656)
**Female**	29707	28674(97)	450	1923	2576	2249	20	19	35	80	76	140
										(48–112)	(45–107)	(97–182)

*Includes population screened by X-ray and /or TB symptoms

# Smr = Smear; Cult = Culture; Bact = Bacteriologically

Imputed datasets: After carrying out multiple imputation for missing data, the overall prevalence (with 95% CIs) of smear, culture and bacteriologically positive TB predicted by the logistic model was 241 (175–332), 265 (202–347), 359 (287–448) per 100,000 population respectively. It was observed that the estimates obtained based on imputed values were slightly higher with wider confidence limits as it allowed for both clustering and the uncertainty involved in imputing the missing values.

Of 3,637 persons with chest symptoms, 306 (8%) had an abnormal radiograph. Of these individuals, 25 (8%) were culture and smear positive, 15 (5%) were culture positive and smear negative, while 6 (2%) were culture negative and smear positive. Among 3,331 X-ray negative chest symtomatics less than 1% were positive by either smear or culture, Table 2.

**Table 2:** Results of symptom screening, chest X-ray, sputum smear, and sputum culture examination.

Results of Screening	Culture postive		Culture negative	
	Smear positive(%)	Smear negative(%)	Smear positive(%)	Smear negative(%)
**Chest Symptoms positive**				
Chest X-ray positive (n = 306)	25(8.0)	15(5.0)	6(2.0)	260(85.0)
Chest X-ray negative (n = 3331)	9(0.3)	18(0.5)	26(0.8)	3278(98.4)
**Chest Symptoms negative**				
Chest X-ray positive (n = 914)	29(3.2)	23(2.5)	10(1.1)	852(93.2)
Chest X-ray negative (n = 45747)	29(3.2)	1(0.002)	1(0.002)	45745(99.996)

Of 46,661 persons without chest symptoms, 914 (2%) had abnormal radiographs. Of these, < 4% were positive by either smear or culture. Among the 45,747 individuals with no symptoms and normal radiographs, 99.9% were negative by smear and culture, Table 2. Notably, of 170 bacteriologically positive TB patients, 64 (38%) were asymptomatic.

### Risk factors associated with culture and bacteriologically positive TB

Univariate analysis using either culture or bacteriologically positive PTB disease resulted in similar findings. Higher odds ratios for both culture and bacteriologically positive PTB was observed among individuals aged ≥ 35 years, men, slum dwellers, those with a past history of TB treatment, BMI ≤ 18.5 Kgs/m^2^, current alcohol users, current tobacco smokers, and those exposed to cooking fuel producing smoke, Table 3. It was also observed that occurrence of culture and bacteriologically positive TB increased with age, the quantity of alcohol consumed, and number of cigarettes/beedis smoked per day, Table 3. However, multivariate logistic regression analysis showed that age ≥ 35 years, BMI ≤ 18.5 Kgs /m^2^, a past history of anti−TB treatment, and being a male current consuming alcohol and using solid cooking fuel were significantly associated with the occurrence of culture and bacteriologically positive TB, after adjusting for factors like gender, smoking, number of cigarettes smoked per day and quantity of alcohol consumed per day, Table 4. While living in a slum was significantly associated with occurrence of bacteriologically positive TB, there was only a borderline significance for occurrence of culture positive TB.

**Table 3:** Univariate analysis of factors associated with the occurrence of bacteriologically (Bact) positive and culture (Cult) positive PTB.

Factors	Population examined	Bacteriologically Positive TB			Culture Positive TB		
		% Bact +ve cases#	OR (95%CI)	p-value	% Cult+ve cases	OR (95%CI)	p-value
**Age**							
15–34	24687	0.1	1	p<0.00001	0.1	1	p<0.00001
35–54	20589	0.4	3.94 (2.52–6.18)		0.3	4.45(2.61–7.61)	
> = 55	9575	0.7	6.53 (4.11–10.39)		0.5	7.01(4.01–12.23)	
**Gender**							
Female	28347	0.1	1	p<0.00001	0.1	1	p<0.00001
Male	26504	0.5	4.14 (2.85–6.01)		0.4	6.04(3.71–9.85)	
**Living conditions**							
Non-slum	38559	0.2	1	p<0.00001	0.2	1	p<0.00001
Slum	16292	0.5	2.26 (1.68–3.06)		0.4	2.30(1.62–3.26)	
**BMI**							
≥18.5	47282	0.2	1	p<0.00001	0.1	1	p<0.00001
<18.5	6310	1.2	6.04 (4.45–8.19)		1	5.63(4.09–7.75)	
**Past History of Treatment**							
No	53702	0.2	1	p<0.00001	0.2	1	p<0.00001
Yes	1148	3.7	15.9 (11.2–22.6)		2.6	15.00(9.9–22.67)	
**Smoking status**							
No	47100	0.2	1	p<0.00001	0.1	1	p<0.00001
Yes	7751	1	5.58 (4.12–7.54)		0.9	7.41(5.22–10.54)	
**No of cigarettes/Beedies smoked per day**							
Nil	47100	0.2	1	p<0.00001	0.1	1	p<0.00001
1–10	5731	0.7	3.90(2.70–5.63)		0.6	5.22(3.43–7.93)	
11–20	1835	2.1	11.47(7.85–16.76)		1.8	15.11(9.82–23.26)	
>20	185	0	0		0	-	-
**Alcohol status**							
No	44917	0.2	1	p<0.00001	0.1	1	p<0.00001
Yes	9934	1	6.36 (4.68–8.63)		0.8	8.79(6.08–12.72)	
**Quantity of alcohol consumed**							
Nil	44917	0.2	1	p<0.00001	0.1	1	p<0.00001
1–100ml	6903	1	6.10(4.36–8.53)		0.8	8.69(5.84–12.92)	
100–500ml	2955	1.1	6.91(4.55–10.51)		0.9	8.90(5.43–14.60)	
>500ml	76	1.3	8.42(1.16–61.41)		1.3	13.91(1.89–102.36)	
**Type of cooking fuel**							
Smokeless	14921	0.4	1	p<0.00001	0.3	1	p<0.00001
Solid	4455	0.9	2.74(1.87–4.02)		0.9	2.87(1.88–4.38)	

*a p-value of <0.05 was considered significant;

#: Bact = Bacteriologically,

Cult = Culture

**Table 4:** Multivariate analysis of factors associated with the occurrence of bacteriologically positive and culture positive PTB.

	Bacteriologically positive TB		Culture positive TB	
Factors	Adj OR (95% CI)	p value*	Adj OR (95% CI)	p value*
**Age**				
15–34	1	0.0002	1	
35–54	3.84(1.64–9.00)		3.81(1.50–9.72)	0.001
> = 55	5.92(2.47–14.2)		5.85(2.24–15.3)	
**Living condition**				
Non-slum	1		1	
Slum	1.6(1.05–2.35)	0.03	1.6 (1.00–2.45)	0.05
**BMI**				
> = 18.5	1			
<18.5	6.05 (3.97–9.22)	<0.00001	8.31(5.22–13.23)	<0.00001
**Past history of treatment**				
No	1			
Yes	5.85(3.62–9.45)	<0.00001	5.33(3.13–9.07)	<0.00001
**Cooking fuel**				
Smokeless	1			
Solid	1.81 (1.20–2.73)	0.005	1.81 (1.15–2.85)	<0.00001
Gender * Alcohol status				
Being a Male and currently consuming alcohol	3.01(1.85–4.89)	<0.00001	3.51(2.02–6.11)	<0.00001

*a p-value of <0.05 was considered significant

### Drug susceptibility pattern

*M*. *tuberculosis* isolates from the 120 culture positive patients were subjected to drug susceptibility testing, of which 96 were new cases and 24 were previously treated for TB. Mono−drug resistance was detected in 8 newly diagnosed patients and 4 previously treated ones. Multi−drug resistance was detected in isolates from 5 newly diagnosed patients cases and in 1 previously treated patient, Table 5.

**Table 5:** Drug sensitivity patterns of PTB cases in Chennai.

Status	New Cases	Previously Treated Cases
	n = 96	n = 24
**Sensitive**	79(82.3)	17(70.8)
**Mono Drug Resistance**		
Isoniazid **(H)**	3(3.1)	1(4.2)
Rifampicin **(R)**	1(1.0)	1(4.2)
Ethambutol **(E)**	1(1.0)	-
Streptomycin **(S)**	3(3.1)	2(8.3)
**Resistance to more than one Drug (S+H,H+E)**	4(4.2)	2(8.3)
**Multi Drug Resistance (H+R, H+R+E, S+H+R+E)**	5(5.2)	5(5.2)

H = Isoniazid, R = Rifampicin, S = Streptomycin, E = Ethambutol

### Spoligotyping results

**Fig 4:**
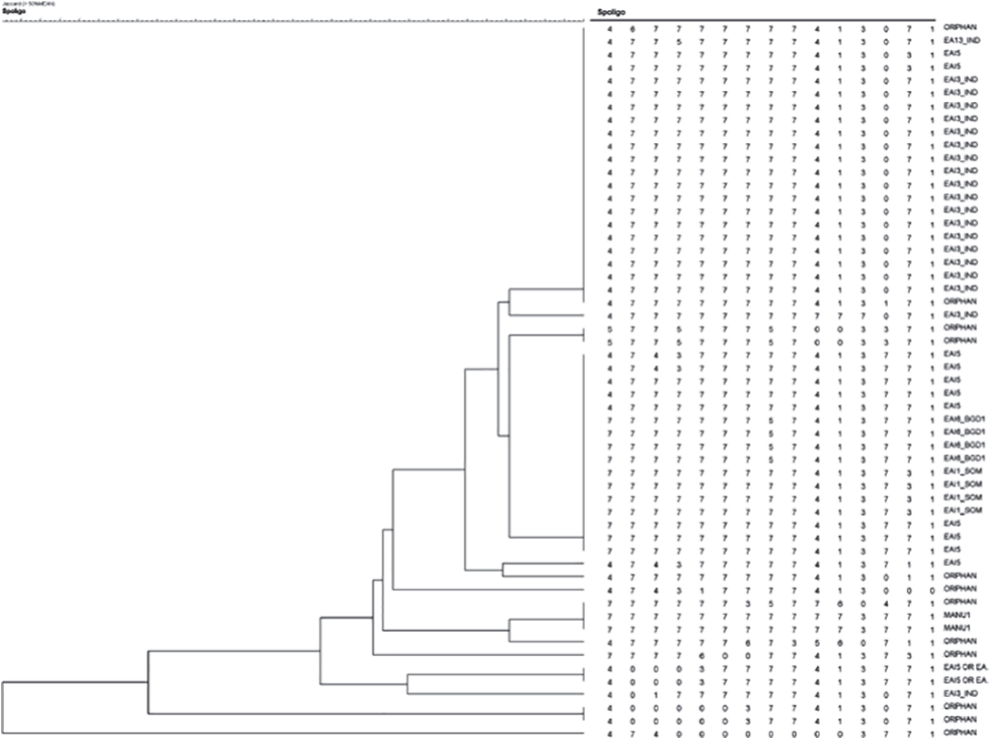
Dendrogramme showing Spoligotyping results among bacteriologically positive pulmonary TB cases in Chennai. Tree presenting the spoligotyping patterns *Mycobacterium tuberculosis* isolates n = profiles by using Bionumerics software with jaccards coefficient. EAI: East African Indian has six subtypes EAI1-6. IND: India, SOM: Somalia, BGD: Bangladesh is represented here. Orphan strains are defined as strains which are not present in the SpolDB4 spoligotype international database of the institute Pasteur de la Guadeloupe.

Spoligotyping generated 22 different patterns as shown in Fig 4. Forty two isolates (78%) were grouped into 10 spoligotype clusters. Further, forty two strains (78%) belonged to a spoligotype described in the international databases (SpolDB4, SITVIT), while 12 (22%) were unidentified. The most frequent spoligotype families were East African Indian (48 isolates), followed by the Manu and T family (6 isolates each). A dendrogram showed 80% similarity between strains, except for 4 strains which showed a degree of variation between 40 to 78%. On matching with the location of patients, no obvious clustering of strains was observed.

### Spatial distribution of pulmonary TB cases

**Fig 5:**
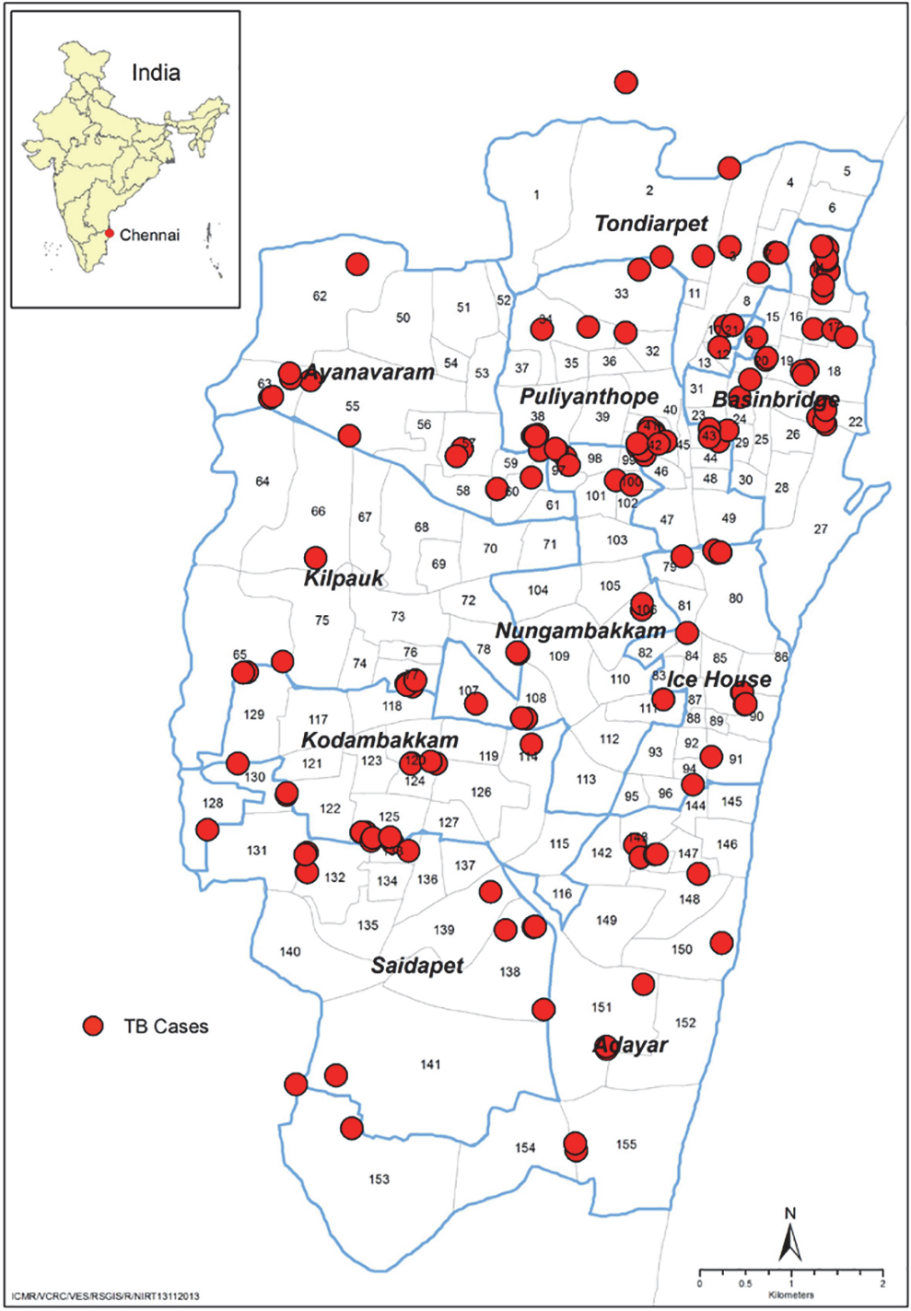
Spatial Distribution of bacteriologically positive TB cases in Chennai.

Spatial distribution of geocoded households with bacteriologically positive PTB cases in Chennai city is shown in Fig 5. It was observed that the northern part of the city had more TB cases compared to the southern part. Further, a comparatively higher number of the PTB patients were seen to reside in the north eastern parts (particularly the zones Tondiarpet, Basin Bridge and Ice House) of Chennai city, which are densely populated areas.

## Discussion

This is one of the first community-based TB prevalence surveys in a large metropolitan city in India, in which the population was screened for both chest symptoms and by mass miniature radiography. The high overall prevalence rates of culture positive, smear positive and bacteriologically PTB among adults of 259 228, and 349/100,000 in Chennai city highlight the need for a renewed urban TB control strategy. This survey reflects better the "true" prevalence of TB disease in an urban population whereas the RNTCP notification data relates predominantly only to those patients attending the public sector health services.

The prevalence estimate of culture positive TB from this study is comparable to the national estimate of 256 [1], but slightly higher when compared to the estimate of 207 from a study conducted in central India [13]. However, compared with the results of earlier studies in the peri-urban areas of Tiruvallur district, the estimate for culture positive PTB from this study was lower (259 versus 388), but with a higher estimate for smear positive PTB (228 versus 180) [3]. A countrywide sample survey conducted by the Indian Council of Medical Research (ICMR) in 1955–58 in Calcutta, Delhi, Hyderabad, Madanapalli, Patna, and Thiruvananthapuram showed that except for Madanapalli, all other areas had higher prevalence of culture positive PTB in urban areas as compared to rural [14]. In recent surveys conducted in predominantly rural populations in southern and central India using similar methodologies, the prevalence of bacteriologically positive PTB was estimated to be 254 and 255 respectively, which were lower than our study estimates of 349 [12,]. The higher prevalence of TB in urban areas could be explained by overcrowding, poor living conditions and other risk factors common in urban slums, as well as delays in diagnosis and suboptimal treatment practices in the private sector.

Age and sex have been observed elsewhere as strong determinants of TB disease, with a higher risk of TB disease observed amongst older individuals and men [15]. Similar observations have been made in four previous TB prevalence surveys conducted in the adjacent Tiruvallur district and numerous other studies conducted in India and other parts of the world [9, 16–18]. The current survey also showed a very high prevalence of PTB disease among men ≥55 years of age. Undiagnosed patients in this group could be contributing to the burden of TB in India. Hence, the national program should develop Information Education and Communication (IEC) strategies focusing specifically on this and other risk groups, to create awareness and encourage people to undergo screening. Training of community health care workers or volunteers for early identification of chest symtomatics in these high-risk groups and referral for diagnosis using more sensitive diagnostics should also be considered.

We observed a greater fall in the prevalence of smear positive PTB compared to culture/ bacteriologically positive PTB amongst those aged ≥65 years. Hence, older patients with active TB may not be diagnosed by sputum smear examination, which is the main tool used for diagnosing PTB under RNTCP. As those ≥65 years of age are less likely to be mobile, one could speculate that these patients could be contributing to disease transmission in their homes, impacting children in particular. Although, we do not have a clear understanding of older age and its role in TB transmission, this group may need targeting with active case finding activities and the use of more sensitive diagnostics.

A recent paper from India found that the population attributable fraction (PAF) for under-nutrition as a risk factor for TB was over 50% [15]. In the present study, a body mass index <18.5Kgs/m^2^ conferred an 8.3 times higher risk of active TB compared to a BMI ≥18.5 Kgs/m^2^. Further, those with a past history of TB treatment were observed to have a 5.3 times higher risk of culture positive TB. A few studies from India have documented relapse rates of 10–12% following treatment of newly diagnosed TB patients [19, 20]. It is possible that the thrice-weekly regimen used under programmatic conditions and if not fully supervised, could lead to high relapse rates. It has also been found that in the presence of baseline isoniazid resistance, intermittent treatment leads to higher failure/relapse [21]. This could explain why previous TB treatment might be a risk factor for current TB. The recently published Indian Standards of TB care now recommends daily treatment for all TB patients in India [22].

Alcohol has been found to have significant inhibitory effects on cell-mediated immunity [23]. Our results show that those men who were currently consuming alcohol, irrespective of the volume consumed, had a 3.5 times higher risk of having culture positive PTB than those not consuming alcohol. This is similar to the findings of an earlier study conducted in Tiruvallur district [18]. However, an age and sex matched case control study carried out among newly diagnosed PTB patients in Bangalore [24]. These findings may reflect regional differences in patterns of alcohol consumption (including differences in alcohol content) or could be due to methodological differences in assessment of the above factors by these studies.

Nicotine is shown to inhibit the production of tumour necrosis factor-alpha by lung macrophages, thereby rendering tobacco smokers more susceptible to progression from latent TB infection to active TB disease [25]. Our study however did not show an association between TB and smoking on multivariate analysis, which is contrary to various other studies which have found an association [18, 26–28]. This may be because of the interaction between smoking, alcohol consumption and male gender.

The study observed that slum dwellers had a 1.6 times higher risk of both culture and bacteriologically positive PTB than non-slum dwellers. Occurrence of TB among slum dwellers is a complex interplay of socioeconomic factors, including factors such as overcrowding, poor personal hygiene, poor ventilation and sanitation. A study conducted among an urban community in Bissau also found an association between poor living conditions as an independent risk factor for developing TB [29]. Those using solid cooking fuel had a 1.8 higher risk of both culture and bacteriologically positive PTB but two matched case control studies conducted in South India and China [24, 30] could not find any association for the same.

More TB cases were distributed among the wards situated in the north part of Chennai, which fall in the zones where the population density is very high[31]. However, as the sample sizes were not sufficiently powered to estimate the ward wise prevalence in the separate zones, we could not carry out appropriate spatial analysis to see if this clustering was significant.

Spoligotyping found that the East African Indian (EAI) strain was the major strain prevalent in this population. A previous study conducted at our institute had also identified the EAI lineage as the major strain circulating in a nearby rural population [32]. Predominant *M*. *tuberculosis* lineages from India and south Asia include EAI, CAS, Beijing and T lineages [33–36]. It is noteworthy that highly transmissible lineages such as Beijing type were absent. The reasons for the observed successful adaptation and predominance of the EAI lineage in this population needs further study.

A limitation of the study was that only selected risk factors were measured and other potential confounders (e.g HIV status, diabetes, income, education, diet etc.) could not be controlled for due to non-availability of information. This might have resulted in an overestimation or underestimation of their true effects. The details of health seeking for symptoms and current TB treatment were also not collected. Further, as the number of isolates tested for drug susceptibility were few in number, it was not possible to make any valid conclusions regarding drug resistance rates.

## Conclusions

The overall estimated prevalence of adult pulmonary TB disease in Chennai city, south India, was high and appeared to be more concentrated in some areas of the city. Whilst men had higher rates than women at all ages, those > 55 years had a prevalence of >1%. Undernutrition, slum dwelling, past history of TB treatment, current consumption of alcohol and use of solid cooking fuel were strongly associated with TB disease. Our findings confirm that TB is a major public health problem in this southern Indian city, and highlights the need for re-thinking current TB control strategies. Future research should study the impact of interventions including deployment of more sensitive diagnostics in certain areas, active case finding in high risk groups, engaging private practitioners in TB control and undertaking an extensive media campaign.
